# D-isoascorbyl palmitate: lipase-catalyzed synthesis, structural characterization and process optimization using response surface methodology

**DOI:** 10.1186/1752-153X-7-114

**Published:** 2013-07-08

**Authors:** Wen-Jing Sun, Hong-Xia Zhao, Feng-Jie Cui, Yun-Hong Li, Si-Lian Yu, Qiang Zhou, Jing-Ya Qian, Ying Dong

**Affiliations:** 1 School of Food and Biological Engineering, Jiangsu University, Zhenjiang 212013, P.R. China; 2 Jiangxi Provincial Engineering and Technology Center for Food Additives Bio-production, Dexing 334221, P.R. China; 3 Parchn Sodium Isovitamin C Co. Ltd, Dexing 334221, P.R. China

**Keywords:** Isoascorbyl palmitate, Enzymatic synthesis, Structural characteristic, Response surface methodology, Optimization

## Abstract

**Background:**

Isoascorbic acid is a stereoisomer of L-ascorbic acid, and widely used as a food antioxidant. However, its highly hydrophilic behavior prevents its application in cosmetics or fats and oils-based foods. To overcome this problem, D-isoascorbyl palmitate was synthesized in the present study for improving the isoascorbic acid’s oil solubility with an immobilized lipase in organic media. The structural information of synthesized product was clarified using LC-ESI-MS, FT-IR, ^1^H and ^13^C NMR analysis, and process parameters for high yield of D-isoascorbyl palmitate were optimized by using One–factor-at-a-time experiments and response surface methodology (RSM).

**Results:**

The synthesized product had the purity of 95% and its structural characteristics were confirmed as isoascorbyl palmitate by LC-ESI-MS, FT-IR, ^1^H, and ^13^C NMR analysis. Results from “one–factor-at-a-time” experiments indicated that the enzyme load, reaction temperature and D-isoascorbic-to-palmitic acid molar ratio had a significant effect on the D-isoascorbyl palmitate conversion rate. 95.32% of conversion rate was obtained by using response surface methodology (RSM) under the the optimized condition: enzyme load of 20% (w/w), reaction temperature of 53°C and D- isoascorbic-to-palmitic acid molar ratio of 1:4 when the reaction parameters were set as: acetone 20 mL, 40 g/L of molecular sieves content, 200 rpm speed for 24-h reaction time.

**Conclusion:**

The findings of this study can become a reference for developing industrial processes for the preparation of isoascorbic acid ester, which might be used in food additives, cosmetic formulations and for the synthesis of other isoascorbic acid derivatives.

## Background

D- isoascorbic acid (synonyms: Erythorbic acid) is a stereoisomer of ascorbic acid (Vitamin C). It is a novel food antioxidant and preservative with excellent safe performance [1]. D- isoascorbic acid can prevent the food oxidation, inhibit the decrease of color, aroma and flavors, and block the production of the carcinogen ammonium nitrite during food manufacturing process. It had been classified as generally recognized as safe (GRAS) additives by US Food and Drug Administration (FDA). Now it can be used in processed foods in accordance with Good Manufacturing Practice (GMP) [2]. D-isoascorbic acid is freely soluble in water. However, its highly hydrophilic behavior similar with ascorbic acid prevents its application in cosmetics or fats and oils-based foods [3]. Esterification process of converting ascorbic acid to its acid esters has been regarded as an effective solution for overcoming such problems. Furthermore, the esterified ascorbic acid products also have bifunctional activity including its original antioxidant activity and the bioactivity of the connected group. For example, the biosynthesized ascorbyl benzoate owned the antioxidant and antimicrobial/ antifungal activities from original ascorbic acid and connected benzoic acid group [4]. And the fatty acid ester of ascorbic acid also has the antioxidant and surfactant functions with its potential application in high-fat food and cosmetics [5-7]. As for the isoascorbic acid, an erythorbyl fatty acid ester of erythorbyl laurate had been recently synthesized for improving the lipophilicity [8]. However, other erythorbyl fatty acid esters are still needed for enlarging its application fields, especially in oil & fat foods.

Oil-soluble ascorbic acid derivatives can be prepared by enzymatic or chemical synthesis [9-11]. For the chemical esterification process, a strongly corrosive acid including hydrogen fluoride or sulfuric acid is used as a catalyst, which results in a series of disadvantages, for example, formation of many side-products and high energy consumption [12]. Enzymatic synthesis is preferred because of its advantages-high catalytic efficiency, mild reaction condition, and inherent selectivity of the natural catalyst [12-15]. As for isoascorbic acid industry, development of its ester products is attractive for enlarging the application fields of oil foods, cosmetics and pharmaceuticals. Furthermore, other erythorbyl fatty acid esters are still needed for increase its application fields. Optimizating the reaction parameters for esterification reaction plays an important role for maximum yield and economical production of isoascorbyl palmitate. Various statistical optimization techniques such as response surface methodology (RSM) with Central Composite Rotatable design (CCRD), Box-Behnken or uniform design method had been applied for ascorbyl palmitate sysnthesis [13], L-ascorbyl laurate [16], ascorbyl oleate [17] and L-ascorbyl lactate [18]. However, there have been no detailed reports on the effects of the reaction parameters on isoascorbic esters production till now.

• The objectives of this study were to: (1) synthesize an oil-soluble isoascorbic acid palmitate by enzymatic method in an organic solvent system, (2) clarify the structural information using LC-ESI-MS, FT-IR, ^1^H and ^13^C NMR analysis, (3) evaluate the key reaction parameter for D-isoascorbyl palmitate process, and (4) optimize the reaction parameters for maximum conversion rate of D-isoascorbyl palmitate using response surface methodology.

## Results and discussion

### Identification of isoascorbic acid and its esters by LC-MS

**Figure 1:**
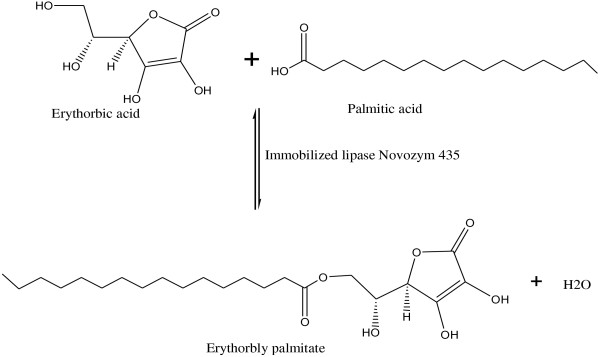
The scheme of lipase-catalysed synthesis of D-isoascorbyl palmitate.

**Figure 2:**
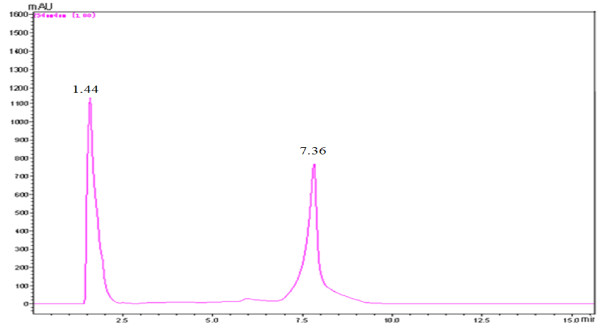
HPLC-PDA chromatogram for the components obtained from the immobilized lipase-catalysed esterification between isoascorbic acid and palmitic acid.

Figure 1 was the schematic diagram of D-isoascorbyl palmitate catalyzed by lipase in organic media. To determine the production yield of the lipase- catalysed esterification between palmitate acid and isoascorbic acid, the wave full scan was conducted at the diode array detector from 180 nm to 1000 nm to select the optimal determining wavelength for the samples. Results showed that the absorbance of isoascorbic acid and isoascorbyl palmitate had the maximum level when the wavelength was set as 254 nm. Thus the HPLC analysis was conducted at the wavelength of 254 nm. Figure 2 showed the HPLC chromatograph of the reaction solution samples with ultraviolet detector. Isoascorbic acid and isoascorbyl palmitate peaks separated well with the retention times of 1.44 and 7.36 min, respectively. The mass spectra of LC-MS indicated that the sample had the mass-to-charge ratios of the molecular ion peak (M -H) of 413.35 and (2M - H) of 827.00, while D-isoascorbyl palmitate should have a mass-to-charge ratio of 414 (M), which proved that the synthesized sample is D-isoascorbyl palmitate

### Structural characteristic analysis of the synthesized D-isoascorbyl palmitate

**Figure 3:**
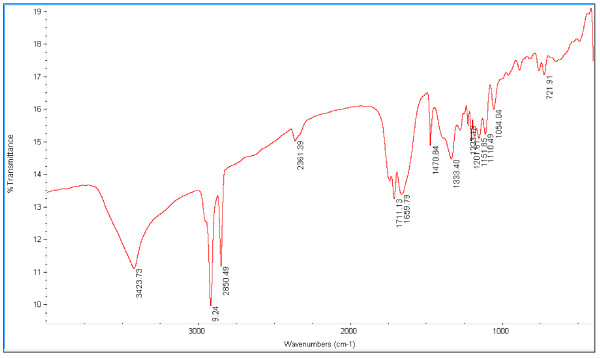
FT-IR spectra of isoascorbyl palmitate synthesized by lipase.

The FT-IR spectrum for the sample isoascorbyl palmitate was presented in Figure 3. The band in the region of 3423 cm^-1^ is due to the hydroxyl stretching vibration. The band in the region of 2930 cm^-1^ and 2850 cm^-1^ are due to C-H stretching vibration in CH_2_ and 1711 cm^-1^ is the absorption of C=O stretching vibration. Absorption at 1659 cm^-1^ was typical for dual bond C = C in isoascorbic acid. The band of 1470 cm^-1^ was characteristic absorption of CH_3_. The characteristic absorption at 1341 cm^-1^, 1225 cm^-1^, 1151 cm^-1^, 1110 cm^-1^ and 1054 cm^-1^ in the FT-IR spectrum was indicative of C-O-C linkage in the isoascorbyl palmitate while the absorption at 721 cm^-1^ also indicated the presence of linked palmitic acid.

**Figure 4:**
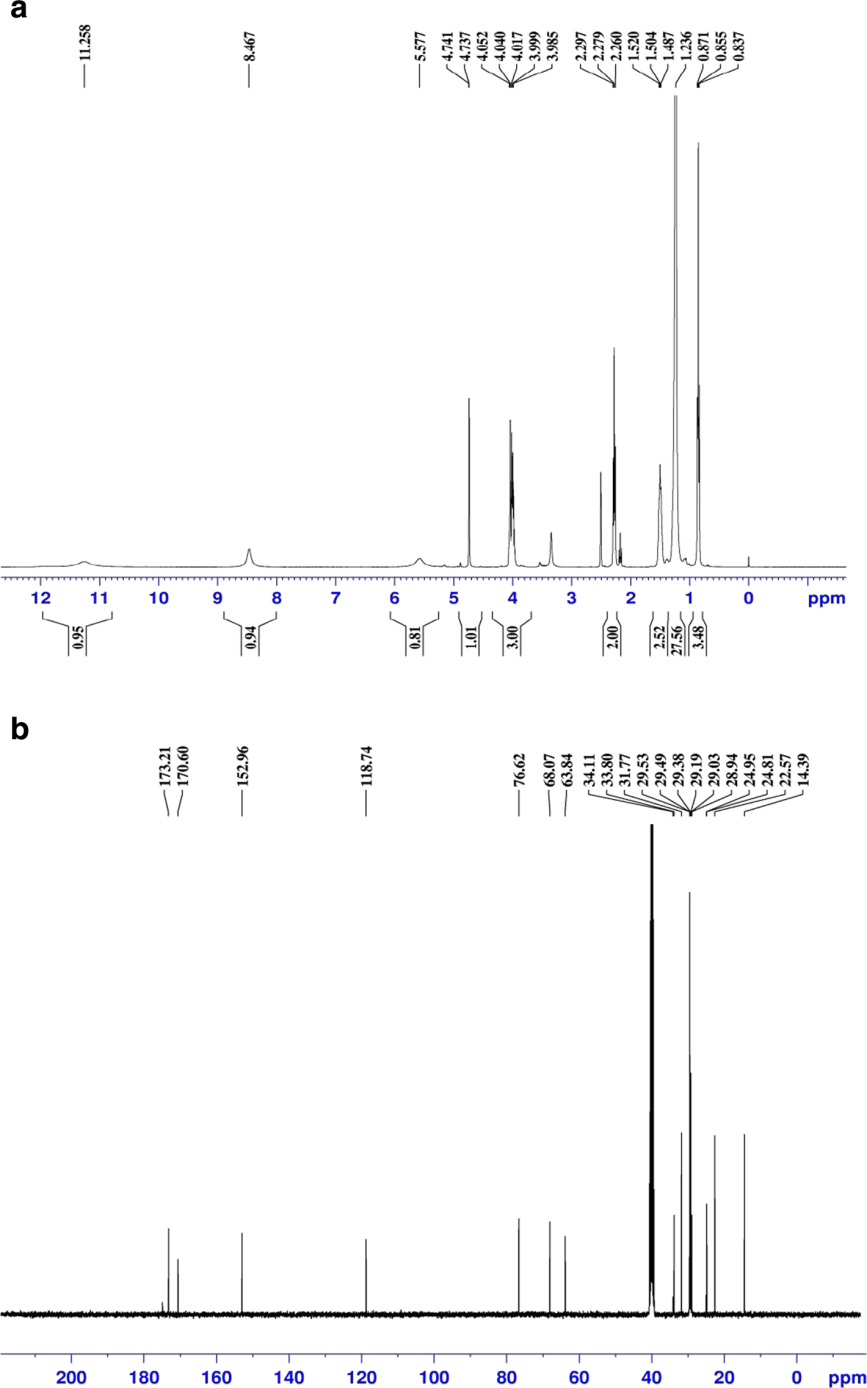
^**1**^**H (a) and **^**13**^**C NMR (b) spectra of isoascorbyl palmitate synthesized by lipase in present study (400 MHz, DMSO-d6).**

^1^H, ^13^C NMR spectra were determined as follows (Figure 4), ^1^H NMR (400 MHz,DMSO-d6):δ (ppm):11.258(s,1H,-OH), 8.467 (s,1H,-OH), 5.577(s,1H,-OH), 4.739 (d,1H,-CH,J=1.6 Hz), 4.017(m,3H,-OCH2-OH), 2.279 (t,2H,-CH2CO,J=7.6Hz), 1.504 (t,2H,-CH2-,J=6.8 Hz), 1.236 (m,2H,12-CH2-), 0.855 (t,3H,-CH3,J=7.2 Hz).

^13^C NMR(400MHz,DMSO-d6):δ (ppm):(173.21 (C-1=O), 170.60 (C-1'=O), 152.96 (C-2), 118.74 (C-3), 76.62(C-4), 68.07(C-5), 63.84(C-6), 34.11 (C-2'), 33.80 (C-3'), 31.77-28.94 (C-4'-C12'), 24.95 (C-13'), 24.81 (C-14'), 22.57 (C-15'), 14.39 (C-16').

The ^13^C NMR spectrum of isoascorbyl palmitate showed the carbonyl group at C-1 and double bonds between C-2 and C-3 in isoascorbic moiety were intact which indicated that the enzymatic reaction happened in other position. The C-6’ signal at 65.6 ppm in the synthesized isoascorbyl ester had a down-field shift of 3.9 ppm in comparison with that of isoascorbic acid (61.7 ppm). These results proved the presence of an ester bond on C-6′of the isoascorbyl moiety and correspond with the pattern of chemical shift reported by Park et al. [8] and Stamatis et al*.*[19].

### One-factor-at-a-time experiments for isoascorbyl palmitate synthesis process

#### Effect of lipase source on D-isoascorbyl palmitate synthesis

Lipases (E.C. 3.1.1.3) generally catalyze the hydrolysis of oils and fats [20,21]. Under specific conditions, they also catalyze the hydrolysis reactions in organic solvents by direct esterification with free acid, transesterification, acidolysis, alcoholysis and aminolysis [22,23]. The lipases sources had the difference in structure including the lid region structure which affected the catalytic activity, regioselectivity and stereoselectivity.

All the lipases used in the present study were listed in Table 1 with their optimum catalytic activities given from the providers. The screening experiments were conducted under a preliminary set of reaction conditions that may not have been the optimum set for all the lipases. In a typical reaction, 150 mg of immobilized derivative was added to the mixture of D-isoascorbic acid: palmitic acid at 1:4 molar ratio using 2-methyl-2-butanol as solvent. Results obtained showed that Novozym 435 had the highest catalytic efficiency with the conversion rate of 41.3% (*m*/*m*), which was in accordance with previous reported results [24,25]. Using RMIM from *Rhizomucor miehei*, had a lower performance of conversion (15.2%). However, other lipases of LVK-H100 and LBK-B400 had no catalytic effect on the D-isoascorbyl palmitate synthesis. Hence, Novozym 435 from *Candida antarctica* was screened as a catalyst for the D-isoascorbyl palmitate lipase-catalyzed synthesis.

**Table 1:** Influence of the lipase source on the synthesis of D- isoascorbyl palmitate

**Lipase**	**Origin**	**Immobilized matrixe**	**Effective temperature (°C)**	**Specific activity**	**Water content**	**Conversion rate (%)**^**a**^
Novozyme 435	*Candida antarctica*	Macroporous acrylic resin	40-60	10,000PLU/g^b^	1-2%	41.30 ± 2.6
Lipozyme TLIM	*Thermomyces lanuginosus*	Silica granulation	55-70	250IUN/g^c^	5%	4.30 ± 1.9
Lipozyme RMIM	*Rhizomucor miehei*	Anionic exchange resin	30-70	5-6BAUN/g^d^	2-3%	15.20 ± 3.5
LVK-H100	*Aspergillus nige*		15-45	20,000U/g		0
LBK-B400	*Aspergillus nige*		25-65	30,000U/g		0

a: Reaction conditions: D-Isoascorbic 2.5 mmol palmitic acid 10 mmol (Molar ratio was 1:4), lipase load: 15% (weight % of substrates), temperature 50°C, tert-amyl alcohol 20 mL, 50 g/L molecular sieve 4 Å and 200 rpm speed for 24 h.

b: PLU is based on a reaction between propyl alcohol and lauric acid.

c: Interesterification Unit ( IUN) is international unit, based on tributyrin assay.

d: Batch Acidolsis Units Novo (BAUN) is based on a reactionbetween high oleic sunflower oil and decanoic acid at 70- 80°C for 60 min.

#### Effect of reaction medium source on D-isoascorbyl palmitate synthesis

A nonaqueous solvent is essential for lipase synthesis of fatty acid esters. A suitable solvent must be able to dissolve sufficient amounts of both the substrates, i.e. D-isoascorbic acid and palmitic acid. The hydrophobicity of the organic solvent significantly influenced the catalytic power of enzyme by changing the three dimensional conformation of protein, and therefore significantly alters conversion and rate [26-28]. The log *P* value, defined as logarithm of the partition coefficient of a given compound in the standard two phase system of octano/water, has been the most commonly used to express solvent effect on the activity and /or stability of enzymes. Differences in solvent log *P* have been widely used to explain their effect on the catalytic activity and enzymes specificity [29]. A series of solvents, such as ethanol, acetone, chloroform, *tert*-amyl alcohol, *n*-hexanol and petroleum ether with the log *P* value from −0.24 to 3.53 were used for D-isoascorbyl palmitate synthesis. The conversion rates of D-isoascorbyl palmitate were shown in Table 2. Among all the solvents, acetone with the log *P* value of −0.23 gave the highest molar conversion (57.8%). A slightly lower performance was achieved in 2-methyl-2-butanol (log *P* = 1.31) (molar conversion = 49.6%). However, ethanol (log *P* = −0.24), chloroform (log *P* = − 2.0), and petroleum ether (log P = −2.62) had no benefits for the proposed reaction. These obtained results were somewhat inconsistent with general reports that solvents with log P < 2 are less suitable for biocatalysis [30,31]. 2-Methyl-2-butanol is a choice as the reaction solvent for ascrobyl palm ester production with a high conversion from 70 to 75%. However, 2-Methyl-2-butanol has the higher price and toxicity comparing with other solvents including acetone [32]. In conclusion, acetone was selected as the reaction medium for the D-isoascorbyl palmitate synthesis in the following experiments.

**Table 2:** Influence of the organic solvent on the synthesis of D- isoascorbyl palmitate

**Solvent**	**Log *P***	**Conversion rate (%)**^**a**^
Ethanol	−0.24	0
Acetone	−0.23	57.8 ± 1.8
2-Methyl-2-butanol	1.31	49.6 ± 2.3
Chloroform	2	0
Petroleum ether	2.62	0
N-hexane	3.53	25.28 ± 3.9

a: D-isoascorbic 2.5 mmol palmitic acid 10 mmol (Molar ratio was 1:4), Novozym 435 load: 15% (weight % of substrates), temperature 50°C, 50 g/L molecular sieve 4 Å and 200 rpm speed for 24 h.

#### Influence of enzyme load on D-isoascorbyl palmitate synthesis

**Figure 5:**
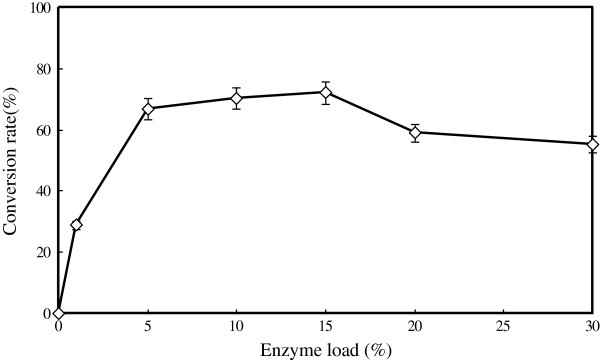
**Effect of enzyme load (weight % of substrates) on lipase-catalyzed synthesis of D-isoascorbyl palmitate.** (Temperature: 50°C; fermentation time: 24 h; molar ratio: 1:4; acetone 20 mL; 4 Å molecular sieves content: 50 g/L; speed: 200 rpm).

The immobilized lipase load volume directly influences the rate and efficiency of the esterification reaction. In the present study, lipase Novozym 435 load varying from 0 to 30% (weight % of substrates) was used (Figure 5). From Figure 5, no D-isoascorbyl palmitate was synthesized when the catalyst Novozym 435 was absent. The convention rate increased from 28.79% to the maximum level of 72.05% with the increase of Novozym 435 load from 1% to 15% (w/w). However, further increase of enzyme (above 15%) load declined conversion ratio to 55.23%. This may be contributed to the high amount of immobilized enzyme was added, especially in the solvent system, the viscosity of the reaction medium was increased and then further led to the less effective transfer of the substrates to the active sites of the excess enzyme molecules inside the bulk of enzyme particles [33,34]. Similar results also were previous reported by Sun et al*.*[35] by obtaining maximum transesterification yield of coconut oil with fusel alcohols when the immobilized lipase TLIM loading volume was 15% (w/w). Thus, immobilized lipase load of 15% w/w) appeared to be the optimal for D-isoascorbyl palmitate synthesis.

#### Effect of reaction time on D-isoascorbyl palmitate synthesis

**Figure 6:**
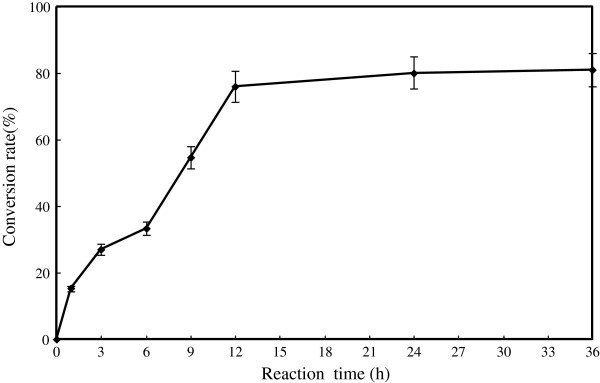
**Effect of time course on lipase catalyzed synthesis of D-isoascorbyl palmitate.** (Enzyme load 15% (weight % of substrates); temperature: 50°C; molar ratio: 1:4; acetone 20 mL; 4 Å molecular sieves content: 50 g/L; speed: 200 rpm).

To check the highest efficiency of D-isoascorbyl palmitate synthesis, the time course of the esterification of D-isoascorbic and palmitic acid catalyzed by the Novozym 435 was monitored. Results were shown in Figure 6. The conversion rate increased rapidly to 80.09% during the 24-h reaction, and then possibly reached to the stable level. For the palm-based ascorbyl esters synthesis, the rapid reaction time was 16-h [32]. Although, maximum conversion ratio of 81% was finally achieved after 36-h synthesis, increase in reaction time also led to a decrease in the reactor working efficiency, which is not economical. For this study, 24-h reaction time was selected.

#### Effect of reaction temperature on D-isoascorbyl palmitate synthesis

**Figure 7:**
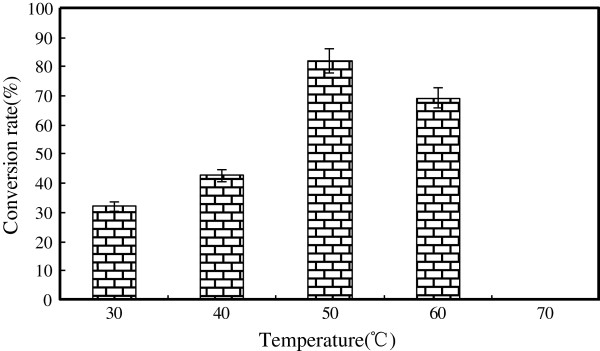
**Effect of temperature on lipase-catalyzed synthesis of D-isoascorbyl palmitate.** (Enzyme load 15% (weight % of substrates); time: 24 h; molar ratio: 1:4; acetone 20 mL; 4 Å molecular sieves content: 50 g/L; speed: 200 rpm).

Reaction temperature had the direct influence of the stability and the activity of the lipase, the solubility of the substrates, the rate of the reaction and the position of the reaction equilibrium [36]. In order to understand the influence of temperature on the D-isoascorbyl palmitate synthesis, the reaction with 2.5 mmol of D-isoascorbic acid and 10 mmol of palmititic acid (Molar ratio was 1:4) loading 15% of Novozym 435 was conducted at five temperatures ranging from 30°C to 70°C (Figure 7). The conversion was significantly affected by the temperature (*P* < 0.01). The maximum conversion rate of 82.05% was obtained at 50°C after 24-h of reaction. The increase of temperature to 60°C inhibited the enzyme catalysis process with the conversion rate of 69.01%. From the Figure 7, Novozym 435 had no catalytic activity when the temperature was set as 70°C with no D-isoascorbyl palmitate production. This result was in consistence with those previously reported that Novozym 435 to be active in nonaqueous systems (organic solvents, solvent-free system, supercritical fluid) at temperatures of 40-60°C [37]. Therefore, 50°C appeared to be the optimal temperature for D-isoascorbyl palmitate production by using Novozym 435 as the catalyst.

#### Effect of substrate molar ratio on D-isoascorbyl palmitate synthesis

**Figure 8:**
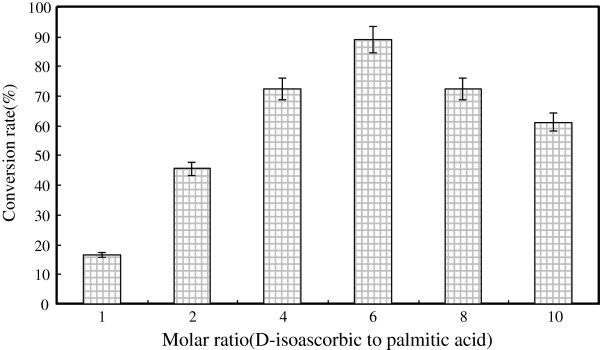
**Effect of molar ratio (D-isoascorbic to palmitic acid) on lipase-catalyzed synthesis of D-isoascorbyl palmitate.** (Enzyme load 15% (weight % of substrates); temperature: 50°C; time: 24 h; acetone 20 mL; 4 Å molecular sieves content: 50 g/L; speed: 200 rpm).

The influence of six substrate molar ratios of D-isoascorbic to palmitic acid, ranging from 1:1 to 1:10 (*m*/*m*), on D-isoascorbyl palmitate production performance was investigated. As shown in Figure 8, the conversion rate increased substantially from 16.66 to 89.21% when substrate molar ratio increased from 1:1 to 1:6 (*m*/*m*) (*P*<0.001). Further increases in molar ratio (beyond 1:6) had no decreased effect on the isoascorbyl palmitate production, which may contribute to the inhibitory effect of high acid concentration on enzyme activity [37]. The same effect was also observed in another study in which oleyl oleate production was investigated using Novozym 435 in a solvent- free system [37,38]. In the present study, substrate molar ratio of 1:6 was optimal for isoascorbyl palmitate production with the highest conversion rate of 84.21%, and used in the following tests.

#### Effect of molecular sieves content on D-isoascorbyl palmitate synthesis

**Figure 9:**
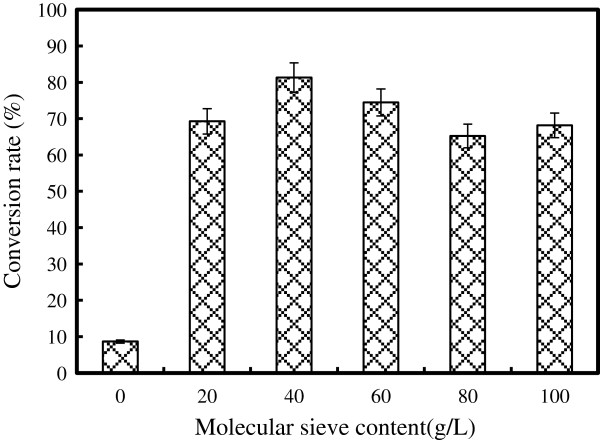
**Effect of molecular sieves on lipase catalyzed synthesis of D-isoascorbyl palmitate.** (Enzyme load 15% (weight % of substrates); time: 24 h; molar ratio: 1:6; acetone 20 mL; temperature: 50°C; speed: 200 rpm)

The ester formation process requires low water content. Lipase catalysis needs a minimal amount of water to ensure its optimal conformation and optimal activity. However, excess of water also negatively decreases the enzyme activity from kinetic and thermodynamic points. Hence, during the lipase-catalyzed synthesis process, removal of the water by using pervaporation or microwave irradiation was unrealistic. Addition of a desiccant such as molecular sieves is an effective method due to the low cost and easy to be separated and regenerated [36]. For the D-isoascorbyl palmitate synthesis reaction, molecular sieves had the use of drying the reaction mixture and adsorbing the produced water to shift the reaction equilibrium. To evaluate the effect of molecular sieves on the conversion performance of isoascorbyl palmitate, 4 Å molecular sieves volume varying from 0 to 100 g/L were added. As shown in Figure 9, lowest conversion rate of 8.64% was obtained without adding 4 Å molecular sieves. A gradual increase up to maximum D-isoascorbyl palmitate conversion rate of 81.31% was observed with the increase of the molecular sieve content to 40 g/L. Further increases in molecular sieves content (beyond 40 g/L) had the negative effect on the isoascorbyl palmitate production. The conversion rate decreased to 65.25% when 4 Å molecular sieves content was 80 g/L. Similar results were also obtained by He et al*.*[39] that the higher molecular sieves concentration up to 80 g/L would result in the lower conversion rate about of 40% by decreasing the activity of lipase. Based on these obtained results, 40 g/L of 4 Å molecular sieves content was used for subsequent experiments in the synthesis of D-isoascorbyl palmitate.

### Response surface optimization

The key parameters including the enzyme load, reaction temperature and molar ration, significantly influencing on the conversion rate of D-isoascorbyl palmitate were obtained based on the “one-factor-at-a-time”(OFAT) experiments, which by changing one factor at a time, and keeping other variables constant. Tables 3 and 4 gave the factors, their values, and the experimental design, respectively. Other reaction parameters are set as follows, 20 mL of acetone 40 g/L of molecular sieves content , 200 rpm of rotation speed for 24-h during the course of optimization experiments. Table 3 showed that the considerable variation in the conversion rate of D-isoascorbyl palmitate under different reaction composition. The isoascorbyl palmitate conversion rate ranged from 37.07% to 93.28%, and the run #10 and #1 had the minimum and maximum ratio values, respectively.

**Table 3:** Variables and experimental design levels for response surface

**Independent variables**	**Coded symbols**	**Levels**		
		−1	0	1
Enzyme load(%, w/w)	A(X_1_)	5	13	20
Temperature(°C)	B(X_2_)	40	50	60
Molar ratio(D-isoascorbic: palmitic acid)	C(X_3_)	2	4	6

**Table 4:** Experimental designs and the results of Box-Behnken design for optimizing reaction conditions for the production of D- isoascorbyl palmitate

**Runs**	**Coded levels**			**Conversion rate (%)**			
	**A**	**B**	**C**	**I**	**II**	**Average**	**Predicted**
1	1(20)	−1(40)	0(4)	92.70	93.86	93.28 ± 0.82	90.33
2	0(13)	1(60)	1(6)	84.78	85.74	85.26 ± 0.68	80.98
3	−1(5)	1(60)	0(4)	62.69	64.63	63.66 ± 1.37	66.61
4	−1(5)	0(50)	1(2)	65.89	66.25	66.07 ± 0.25	67.40
5	0(13)	0(50)	0(4)	84.03	84.75	84.39 ± 0.51	84.66
6	1(20)	0(50)	−1(2)	86.89	85.53	86.21 ± 0.96	84.88
7	1(20)	0(50)	1(6)	85.78	87.46	86.62 ± 1.19	86.91
8	0(13)	−1(40)	−1(2)	50.00	50.60	50.30 ± 0.42	54.58
9	0(13)	1(60)	−1(2)	70.98	71.38	71.18 ± 0.28	68.53
10	−1(5)	0(50)	−1(2)	37.08	37.06	37.07 ± 0.01	36.78
11	0(13)	−1(40)	1(6)	72.88	71.36	72.12 ± 1.07	74.78
12	−1(5)	−1(40)	0(4)	53.89	55.75	54.82 ± 1.32	50.83
13	1(20)	1(60)	0(4)	90.22	91.24	90.73 ± 0.72	94.72
14	0(13)	0(50)	0(4)	83.97	86.01	84.99 ± 1.44	84.66
15	0(13)	0(50)	0(4)	85.02	84.20	84.61 ± 0.58	84.66

#### Model fitting

Table 5 showed the analysis of variance (ANOVA) for this experiment, and the coefficient of determination (R^2^) was shown as 97.34%. This indicated that, the accuracy and general ability of the polynomial model was good, analysis of the response trends using the model was considered to be reasonable. A precision ratio of 15.79 indicates an adequate signal. A ratio greater than 4 is desirable. The relatively low coefficient of variation value (CV=6.15%) indicated the good precision and reliability. The regression coefficients, along with the corresponding *P*-values, for the model of the conversion rate of isoascorbyl palmitate, were presented in Table 5. The *P*-values are used as a tool to check the significance of each coefficient, which also indicate the interaction strength between each independent variable. The smaller the *P* values, the bigger the significance of the corresponding coefficient [40]. Table 5 showed that the quadratic model was highly significant (*p*<0.01). Meanwhile the lack-of-fit the *P* values of 0.0027 indicated that the lack of fit was significant. Enzyme load and molar ratio of D-isoascorbic to palmitic acid had a highly linear effect at 1% level. Temperature was also significant at 5% level. While the interaction effects of independent variables were found no significant quadratic effect (p-value: AB=0.2665, BC=0.4343).

**Table 5:** Results of ANOVA analysis of a full second-order polynomial model for reaction conditions for the production of D- isoascorbyl palmitate

**Source**	**Sum of squares**	**df**	**Coefficient estimate**	***F*-Value**	***P*-Value**
Model	3798.88	9	422.10	20.35	0.0020**
A	2285.56	1	2285.56	110.17	0.0001**
B	203.11	1	203.11	9.79	0.0260*
C	533.17	1	533.17	25.70	0.0039**
AB	32.43	1	32.43	1.56	0.2665
AC	204.35	1	204.35	9.85	0.0257*
BC	14.98	1	14.98	0.72	0.4343
A^2^	87.99	1	87.99	4.24	0.0945*
B^2^	63.87	1	63.87	3.08	0.1397
C^2^	429.81	1	429.81	20.72	0.0061**
Residual	103.73	5	20.75		
Lack of fit	103.55	3	34.52	374.63	0.0027**
Pure error	0.18	2	0.092		
Cor total	3902.61	14			
R-squared	= 0.9734	Adj-Squared	= 0.9256	C.V.% =	6.15

** Significant at 1% level * Significant at 5% level Adeq Precision=15.9.

Using the designed experimental data (Table 3), the polynomial model for conversion rate (%) Y _conversion rate_ was regressed by only considering the significant terms and was shown as below:

(2) $$\begin{array}{ll} Y_{\mathrm{conversion}\;\mathrm{rate}}= & 84.66+16.90X_{1}+5.05X_{2}\\ & +8.16X_{3}-7.15X_{1}X_{3}-1.94X_{2}X_{3}\\ & -4.88X_{1}{}^{2}-10.79X_{3}{}^{2} \end{array}$$

**Figure 10:**
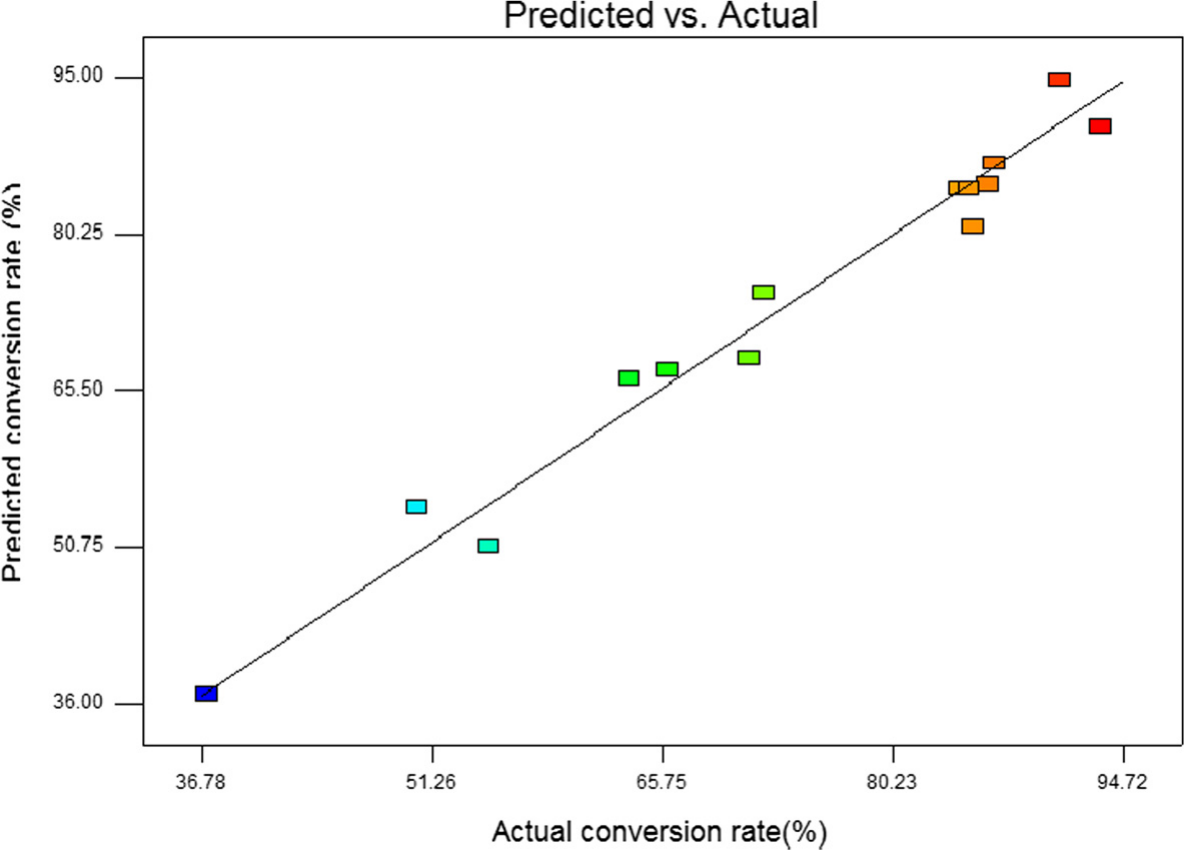
Plot of predicted and observed conversion rate (%) of D-isoascorbyl palmitate.

Where *Y* is the response variable (isoascorbyl palmitate conversion rate, %), and *X*_1_, *X*_2_ and *X*_3_ are enzyme load, temperature and molar ratio of D-isoascorbic to palmitic acid, respectively. Figure 10 shows the observed and predicted conversion rate determined by the model Eq. (2) which indicated an excellent agreement between actual and predicted responses.

#### Mutual effect of parameters and attaining optimum condition

**Figure 11:**
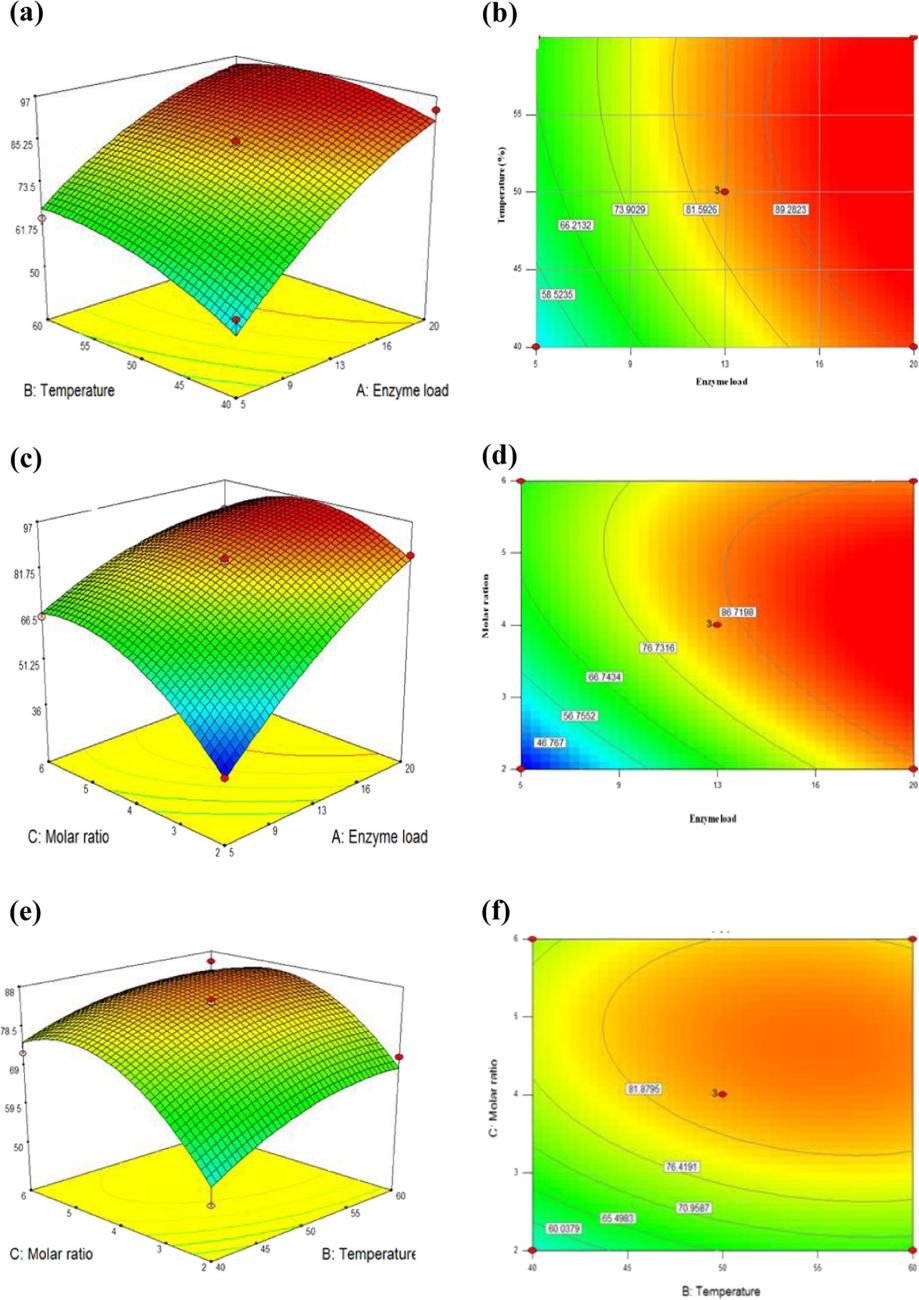
Response surface and 3D contour plots indicating the effect of interaction between reaction parameters on D- isoascorbyl palmitate conversion rate (a, b) interaction between enzyme load and temperature while holding molar ratio at 4 (c, d) interaction between enzyme load and molar ratio while holding temperature of 50°C (e, f) interaction between temperature and molar ratio while holding enzyme load at 13% (w/w).

The response surface and contour plots in Figure 11 show the main, interaction, and quadratic effect of 2 independent variables on conversion rate. Figure 11(a, b) shows the effect of enzyme load (X_1_) and temperature (X_3_) on the conversion rate of isoascorbyl palmitate at the molar ratio kept at a constant level. It was obvious that the conversion rate of isoascorbyl palmitate was sensitive even when enzyme load was subject to small alteration. An increase in the conversion rate could be significantly improved with the increase of enzyme load and temperate. Figure 11(c, d) shows the effect of enzyme load (X_1_) and molar ratio (X_2_) on the conversion rate of isoascorbyl palmitate at the temperature kept at a constant level. From the figures, we can see that a higher conversion rate will obtained using a high molar ratio of D-isoascorbic to palmitic acid. Figure 11(e, f) shows the interaction between molar ratio (X_2_) and temperature (X_3_) on the conversion rate of isoascorbyl palmitate at the enzyme load kept at a constant level. As it is shown, the conversion rate increased slightly with increasing temperature from 40°C to 60°C in the range of molar ratio. The optimal conditions for D- isoascorbyl palmitate synthesis was obtained by using the Point prediction function of software Design-Expert 7.1.1 to calculate maximum level of conversion rate. The maximum conversion rate of D- isoascorbyl palmitate was 96.98% under the reaction conditions as follows: enzyme load of 20% (w/w), reaction temperature of 53°C and D- isoascorbic-to-palmitic acid molar ratio of 1:4.

#### Validation of the model

The availability of the regression model (Eq. (2)) of the conversion rate of isoascorbyl palmitate was tested using the calculated optimal condition, viz. acetone 20 mL, 40 g/L of molecular sieves content, 200 rpm speed, 20% enzyme load, D- isoascorbic-to-palmitic acid molar ratio of 1:4, temperature of 53^o^ for 24-h during the course of optimization experiments. The mean value of the mycelial biomass was 95.32 ± 0.17%, which agreed with the predicted value (96.98%) well that indicated the high validity and adequacy of the model.

## Experimental

### Materials

D-isoascorbic acid (purity > 99%) was provided from Parchn Sodium Isovitamin C Co., Ltd (Dexing, Jiangxi, China). Palmitic acid (purity > 99.5%) was obtained from Sinopharm Chemical Reagent Co., Ltd (Shanghai, China). Novozym 435 was purchased from Novo Nordisk Co., Ltd (Beijing, China). Lipozyme TLIM, a lipase from *Thermomyces lanuginosus* immobilized on silica granulation and Lipozyme RMIM, a lipase from *Rhizomucor miehei*, immobilized on an anionic exchange resin, also purchased from Novo Nordisk Co., Ltd (Beijing, China). Lipase LVK-H100 and LBK-B400, were kindly gifted by Leveking bio-engineering Co., Ltd (Shenzhen, China). The properties of all lipases are shown in Table 1.

2-Methyl-2-butanol, n-hexane, ethanol, chloroform, petroleum ether, acetone and acetic ether were analytical reagent grade purchased from Sinopharm Chemical Reagent Co., Ltd (Shanghai, China). HPLC-grade methanol was purchased from Tedia, USA. All reagents were dehydrated by molecular sieve 4 Å (Shanghai world molecular sieve Co., Ltd., Shanghai, China) for at least 24 h and filtered using a membrane filter (0.45 μm) prior to use as a reaction medium.

### Procedure for lipase-catalysed esterification

D-isoascorbic acid (2.5 mmol), palmitic acid (10 mmol) and the immobilized lipase (150 mg, about 5% of the substrates amount) were weighed into a 150 mL conical flask. 20 mL of 2-methyl-2-butanol and 1.0 g of molecular sieve 4 Å were then added. The stoppered flasks were shaken at the speed of 200 rpm on a thermo-constant orbital shaker at 50°C for 48 h. The sampled reaction mixture was filtered through a membrane filter (0.45 μm), and 20 μL of each aliquot were injected into the HPLC for further analyzing concentrations of the substrate isoascorbic acid and the produced D-isoascorbyl palmitate.

### Purification of produced D- isoascorbyl palmitate

The purification process was conducted according to the method described by Park et al. [8] and Bradoo et al. [41] with a slight modification. Briefly, the reaction solution was filtered with a membrane filter (0.45 μm) to remove the lipase and molecular sieve. The mixture solution of D-isoascorbyl palmitate, isoascorbic acid and palmitic acid was obtained by vacuum evaporating the 2-Methyl-2-butanol, and resolved in ethyl acetate. The same quota of deonized water was added for removing the residue isoascorbic acid, and hexane was used to washing out the palmitic acid. The insoluble D- isoascorbyl palmitate was then finally obtained by vacuum drying for 2 h.

### Structural analysis

Produced D-isoascorbyl palmitate and residual isoascorbic acid was identified by mass spectrometry with a quadrupole ion trap Thermo Finnigan™ LXQ™ LC-ESI-MS (San Jose, CA, USA) equipped with a degasser, LC-20AD binary pumps, a model SIL-20AC autosampler, a model CTO-20A thermostat, an electro-spray ionization (ESI) interface, and a model CBM-20A system controller. FT-IR spectra with Thermo-Nicolet Nexus 670 Fourier Transform Infrared Spectrometer (San Jose, CA, USA), ^1^H and ^13^C NMR spectra with a Bruker AVANCE NMR Spectrometer (Switzerland) at 400 MHz.

### Products quantification

Produced D-isoascorbyl palmitate and residual isoascorbic acid were quantitatively analyzed by using a Waters Alliance LC-20AT (SHIMADZU, Japan) liquid chromatography connected to a model 2996 (DAD) diode array detector and controlled by LC Driver Ver.2.0 for Waters Empower™ software. The column equipped in the HPLC system was ZORBAX Eclipse XDB-C18 (150 mm×4.6 mm, 5 μm, Torrance, CA, USA). The mobile phase was methanol/water (90:10, v/v) at 1.0 ml/min flow rate for 15 min. Samples of 20 μL were injected automatically. The purity of sample was 95% with a sole peak in the HPLC chromatograph, which could be used as a standard. Purified D-isoascorbyl palmitate had the purity of 95% determining with HPLC (data not shown) as the standards (0.2, 0.5, 1.0, 1.5, 2.0, and 2.5 g/L) were used to obtain the D-isoascorbyl palmitate calibration curve. The conversion rate (%) was calculated by dividing the initial molar amount of D-isoascorbic acid by the produced molar amount of isoascorbyl palmitate.

### Experimental design and evaluation

According to the results of “one–factor-at-a-time” experiments, which vary only one factor or variable at a time while keeping others fixed, a response surface methodology (RSM) was used to influence of enzyme load (w/w), temperature and molar ration (D-isoascorbic : palmitic acid) on the conversion rate of the D-isoascorbyl palmitate by lipase-catalyzed synthesis. A three factors, three levels Box-Behnken factorial design was used for fitting a second order response surface, using the software Design Expert 7.1.1 (Stat-Ease, Minneapolis, MN, USA). All other factors, for example reaction time, molecular sieves content were maintained constant. A mathematical model, describing the relationships between the process indices (the conversion rate of D-isoascorbyl palmitate) and the medium component contents in second order equation, was developed. The conversion rate of D-isoascorbyl palmitate was multiply regressed with respect to the reaction parameters by the least squares method as follow:

(1) $$Y=A_{0}+\sum A_{i}X_{i}+\sum A_{\mathrm{ii}}X_{i}^{2}+\sum A_{\mathrm{ij}}X_{i}X_{j}$$

Where Y is the predicted response variable (conversion rate, %); A_o_, A_i_, A_ii_, A_ij_ are constant regression coefficients of the model, and X_i_, X_j_ (i=1, 3; j=1, 3, i≠j) represent the independent variables (reaction parameters) in the form of coded values. The accuracy and general ability of the above polynomial model could be evaluated by the coefficient of determination R^2^.

## Conclusions

Isoascorbyl palmitate was successfully synthesized by using lipase-catalysed esterification of isoascorbic acid and palmitic acid under the mild reaction conditions. It structure was characterized by LC-MS, FT-IR, ^1^H, and ^13^C NMR. The effect of various parameters on synthesis of D-isoascorbyl palmitate, such as enzyme source, type of organic, enzyme load, reaction time, temperature, molecular sieves content and D-isoascorbic-to-palmitic acid molar ratio were discussed using “one–factor-at-a-time” experiments and Response surface methodology. The optimized condition was obtained as follow: enzyme load of 20% (w/w), reaction temperature of 53°C and D-isoascorbic-to-palmitic acid molar ratio of 1:4. Under these optimal conditions, 95.32% of conversion rate was obtained which was in agreement with the predicted value (96.98%). The results are of a reference for developing industrial processes for the preparation of isoascorbic acid ester, which might be used in food additives, cosmetic formulations and for the synthesis of other isoascorbic acid derivatives.

## Competing interests

The authors declare that they have no competing interests.

## Authors^’^ contributions

W-JS and F-JC conceived of the study, participated in its design and coordination, and drafted the manuscript. H-XZ performed experiments and analyzed results and helped to draft the manuscript. Y-HL helped to do experiments. QZ, S-LY, J-YQ and YD performed partial experiments and analyzed results. All authors read and approved the manuscript.
